# Draft Genome Sequences of *Campylobacter jejuni* Strains That Cause Abortion in Livestock

**DOI:** 10.1128/genomeA.01324-16

**Published:** 2016-12-01

**Authors:** Allison M. Weis, Kristin A. Clothier, Bihua C. Huang, Nguyet Kong, Bart C. Weimer

**Affiliations:** a School of Veterinary Medicine, UC Davis, Davis, California, USA; b 100K Pathogen Genome Project, UC Davis, Davis, California, USA; c California Animal Health and Food Safety, Davis, California, USA

## Abstract

*Campylobacter jejuni* is an intestinal bacterium that can cause abortion in livestock. This publication announces the public release of 15 *Campylobacter jejuni* genome sequences from isolates linked to abortion in livestock. These isolates are part of the 100K Pathogen Genome Project and are from clinical cases at the University of California (UC) Davis.

## GENOME ANNOUNCEMENT

*Campylobacter jejuni* is transmissible between wildlife, livestock, and humans, often leading to foodborne illness in humans and disease burden among livestock (1–4). Globally, *C. jejuni* is a common foodborne pathogen (5) that infects over 1.3 million people each year in the United States (6), causing gastroenteritis; in rare cases, it may induce Guillain-Barré syndrome, an autoimmune disease (7–10). In addition to human infection, *C. jejuni* infects domesticated livestock, including sheep, cattle, goats, and pigs, most often leading to gastroenteritis in these species (11, 12).

*Campylobacter* spp., specifically, *C. fetus* subsp. *fetus*, are one of the leading causes of abortion in ungulates, characterized by late term abortion, stillbirths, and occasional ewe deaths (13). In recent years, many cases have recovered *C. jejuni* from aborted fetuses with similar disease pathologies (14, 15). Emergence of abortive hypervirulent *C. jejuni* isolates have been observed in various regions of the United States (14, 15). We sequenced 15 *C. jejuni* isolates associated with abortion in sheep, cows, and goats in northern California at an average coverage of 91×, assembled, and annotated (Table 1).

**TABLE 1:** Coverage and accession numbers of 15 abortive *C. jejuni* genomes

GenBank accession no.	SRA accession no.	Isolate name	Coverage (×)
MJZI00000000	SRR3619957	BCW_6919	93
MKAC00000000	SRR3619958	BCW_6920	91
MKAD00000000	SRR3619959	BCW_6921	79
MKAE00000000	SRR3619960	BCW_6922	100
MKAF00000000	SRR3619963	BCW_6924	90
MKAG00000000	SRR3619964	BCW_6925	84
MKAR00000000	SRR3619965	BCW_6926	84
MKAS00000000	SRR3619966	BCW_6927	80
MKAT00000000	SRR3619967	BCW_6928	50
MKAU00000000	SRR3619968	BCW_6929	76
MKAV00000000	SRR3619969	BCW_6930	101
MKIC00000000	SRR4020196	BCW_6931	111
MKID00000000	SRR4020197	BCW_6932	70
MKIB00000000	SRR4020198	BCW_6933	81
MKIA00000000	SRR4020199	BCW_6934	182

All *C. jejuni* isolates were cases from the University of California (UC) Davis California Animal Health and Food Safety Laboratory System (CAHFS) and sequenced by the 100K Pathogen Genome Project (http://www.100kgenomes.org) in the laboratory of Bart Weimer (UC Davis, Davis, CA). As described (16), isolates were checked for purity (17), genomic DNA (gDNA) was extracted from cultures grown on 5% blood agar plates (UC Davis, VetMed Biological Services) for 1 to 2 days, lysed (18), purified with Qiagen QIAamp DNA minikit, and analyzed on Agilent 2200 TapeStation system using the Genomic DNA ScreenTape assay for integrity of gDNA (19). Isolated gDNA was used to construct libraries using the Kapa HyperPlus kit (KR1145 version 3.16; Kapa Biosystems, Wilmington, MA, USA) with dual-SPRI size selection (20). Libraries were constructed using the PerkinElmer Sciclone next-generation sequencing (NGS) Workstation (PerkinElmer, Hopkinton, MA). Library quantitation was done using the Kapa SYBR Fast quantitative PCR (qPCR) kits (Kapa Biosystems) to ensure the starting concentration of 400 ng and a fragment insert size between 350 and 450 bp (20). Libraries were indexed using Integrated DNA Technologies Weimer 384 TS-LT DNA barcodes, which allowed multiplexing up to 384 isolates. Sequencing was done at the UC Davis Genome Center (Davis, CA, USA) on the HiSeq 3000 instrument using a paired-end 150 protocol (Illumina, Inc., San Diego, CA, USA) (21, 22). Paired-end reads were assembled using ABySS 1.5.2 using *k* = 64 (23).

### Accession number(s).

These sequences can be found in the 100K Project BioProject at the NCBI SRA BioProject PRJNA186441 and in the NCBI GenBank. Individual GenBank and SRA accession numbers are presented in Table 1.
